# An intrinsic association between olfactory identification and spatial memory in humans

**DOI:** 10.1038/s41467-018-06569-4

**Published:** 2018-10-16

**Authors:** Louisa Dahmani, Raihaan M. Patel, Yiling Yang, M. Mallar Chakravarty, Lesley K. Fellows, Véronique D. Bohbot

**Affiliations:** 1 Department of Psychiatry, Douglas Mental Health University Institute, McGill University, Montreal, QC H4H 1R3 Canada; 2 Department of Biological and Biomedical Engineering, McGill University, Montreal, QC H3A 2B4 Canada; 3 Department of Neurology and Neurosurgery, Montreal Neurological Institute, McGill University, Montreal, QC H3A 2B4 Canada

## Abstract

It was recently proposed that olfaction evolved to aid navigation. Consistent with this hypothesis, olfactory identification and spatial memory are linked to overlapping brain areas which include the orbitofrontal cortex and hippocampus. However, the relationship between these two processes has never been specifically investigated. Here, we show that olfactory identification covaries with spatial memory in humans. We also found that the cortical thickness of the left medial orbitofrontal cortex, and the volume of the right hippocampus, predict both olfactory identification and spatial memory. Finally, we demonstrate deficits in both olfactory identification and spatial memory in patients with lesions of the medial orbitofrontal cortex. Our findings reveal an intrinsic relationship between olfaction and spatial memory that is supported by a shared reliance on the hippocampus and medial orbitofrontal cortex. This relationship may find its roots in the parallel evolution of the olfactory and hippocampal systems.

## Abstract (web summary)

Olfaction, the sense of smell, may have originally evolved to aid navigation in space, but there is no direct evidence of a link between olfaction and navigation in humans. Here the authors show that olfaction and spatial memory abilities are correlated and rely on similar brain regions in humans.

## Introduction

Recently, it was suggested that the primary function of olfaction is navigation, a hypothesis termed the olfactory spatial hypothesis^1^. There is evidence that olfaction and navigation are two processes that may be linked: the size of the olfactory bulb, or its equivalent, covaries with hippocampal size in many mammals^2^ and with navigational demand in many animals, including mammals, birds, reptiles, fish, and arthropods^1^. This is particularly striking given that all animals use chemical cues to navigate, find food, or avoid predation, while vision and audition are less common and not present in all animals^1,3^. Together, these pieces of evidence indicate that navigation and olfaction may be intrinsically linked. If this is the case, we would expect olfactory performance to covary with navigation ability.

When it comes to navigation, there are different strategies that can be used to learn to find our way, and these strategies are supported by distinct memory systems. The spatial memory strategy, which involves learning the relationships between the landmarks in the environment and building a cognitive map^4^, relies on a network of areas that includes the hippocampus and the medial orbitofrontal cortex (mOFC)^5–7^. The stimulus-response strategy, on the other hand, is a habit-based form of navigation and involves making stimulus-response associations^8^, such as learning a series of motor actions in response to stimuli (e.g., turn left at the brown building). This strategy develops when one routinely travels the same route^6,9^, and critically relies on the caudate nucleus^8,10^. Thus, navigation is a broad process that includes two distinct strategies or categories, one being spatial learning and memory (spatial memory strategy) and the other being stimulus-response learning and memory (stimulus-response strategy).

We hypothesized that olfactory identification would be specifically associated with the spatial memory strategy and not the stimulus-response strategy. Animals who learn to navigate in a new environment are more likely to use a spatial memory strategy^11^, possibly because learning the relationships between different cues (olfactory or otherwise) and landmarks allows for more flexible behavior, and thus more adaptive responses, compared to stimulus-response navigation which is rigid in nature^4^. Integrating olfactory or chemosensory information into a cognitive map is a crucial step for most animals, for example when learning where food is located. There is already some evidence that olfactory identification may be associated with spatial memory, as both processes appear to involve similar brain regions, which amongst others include the hippocampus and orbitofrontal cortex^5–7,12–18^. However, this has not been tested directly.

In two studies, we sought direct evidence that olfactory identification and spatial memory are related to one another, and tested the hypothesis that such a relationship reflects shared neural substrates. In the first study, we investigated the relationship between olfactory identification and navigation, and found olfactory identification to be specifically associated with spatial memory. We additionally found that structural measures of the mOFC and hippocampus are associated with olfactory identification and spatial memory. The critical role of the hippocampus in spatial memory^16,19–21^ and olfaction^17,18,22,23^ has already been demonstrated in human lesion studies. The mOFC, while critical to olfaction^18,24,25^, has not yet been demonstrated to be critical to spatial memory. Therefore, in the second study, we sought to fill this gap in the literature by testing the critical role of the mOFC in both olfactory identification and spatial memory, and found patients with mOFC damage to exhibit impairments in both processes.

## Results

### Olfactory identification and spatial memory are correlated

In a neuroimaging study, we assessed olfactory identification, navigation, and measured mOFC cortical thickness and hippocampal volumes in 57 healthy young adult participants. To evaluate navigation, we used two tasks. One is a wayfinding task^26^, which measures the ability to build and use a cognitive map of a virtual environment containing landmarks (e.g., pool, shops, etc.), and which therefore requires using spatial memory. This type of spatial memory task has been shown to rely on the hippocampus^9^. To assess whether olfactory identification is specifically associated with spatial memory and not all forms of navigation, we also included a dual-solution task, called the 4-on-8 virtual maze (4/8 VM), which can be solved using either a spatial memory or stimulus-response strategy in the same sample of participants. We hypothesized in this study that olfactory identification would be associated with spatial—and not stimulus-response—learning and memory, and that only spatial learners would show an association between navigation, olfactory identification, and mOFC and hippocampal structure measures. Details on the analyses are described in the Methods section.

**Fig. 1:**
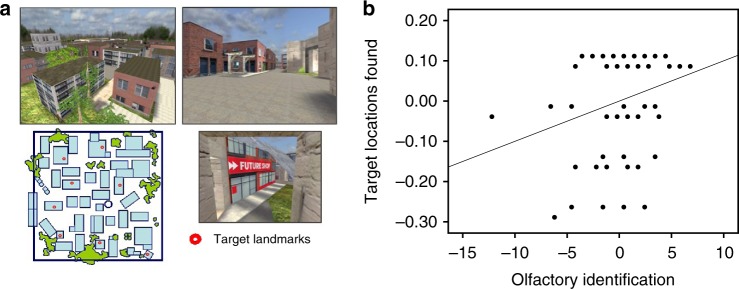
Olfactory identification correlates with wayfinding ability. **a** The wayfinding task involves learning the location of eight key landmarks in a virtual town. Participants first explore the town for at least 20 min, until they have visited every street and alley and passed by each key landmark twice. Then, in eight probe trials, participants are placed in front of one landmark and are asked to find the most direct route to another given landmark. **b** We found olfactory identification to be positively correlated with the percentage of target locations found (*r* = 0.26, Bootstrap BCa 95% CI [0.06, 0.45])

The correlation between olfactory identification task and wayfinding (Fig. 1a) performance was first tested. We found a significant positive correlation between olfactory identification and the percentage of target destinations found, controlling for sex (*r* = 0.26, Bootstrap BCa 95% CI [0.06, 0.45]) (Fig. 1b) and a significant negative correlation with path tortuosity, a measure of path indirectness (*r* = −0.26, Bootstrap BCa 95% CI [−0.43, –0.08]). These findings suggest that the two processes are indeed related; better olfactory identification is associated with better spatial memory, as measured by wayfinding ability.

**Fig. 2:**
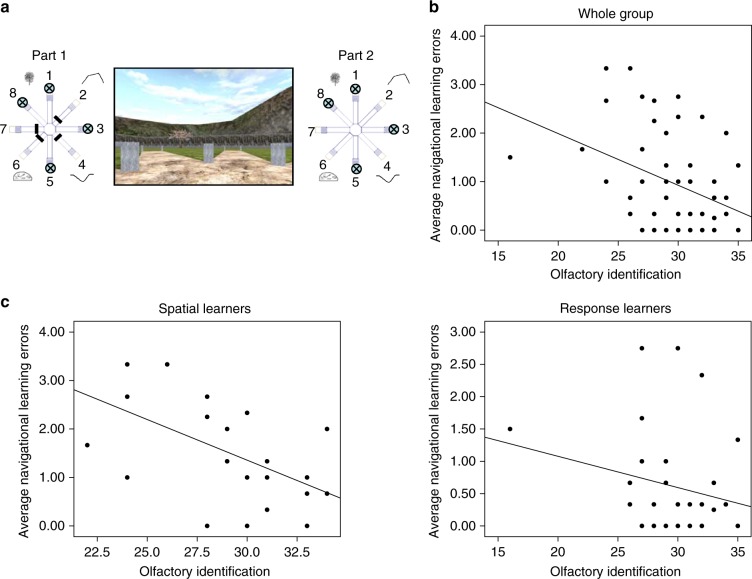
Olfactory identification correlates with spatial memory. **a** The 4-on-8 Virtual Maze (4/8 VM) consists in an 8-arm radial maze surrounded by landmarks. In Part 1, four of the paths are blocked and four are open. Participants have to retrieve objects at the end of the open paths. In Part 2, the barriers are removed. Participants have to avoid the paths they visited in Part 1 to retrieve the remaining objects. They can learn the object locations using a spatial memory strategy (e.g., “the path is to the left of the boulder”) or a stimulus-response strategy (“From the starting position, I have to take the path straight ahead and then skip a path on the right”). Once participants learn the task to criterion, they are taken to a probe stage, where a wall is raised around the maze which hides the landmarks. People who used a spatial memory strategy during learning ('spatial learners') make more errors than people who used a stimulus-response strategy ('response learners'), as they can no longer use landmarks to find the target paths. At the end of the task, participants complete a verbal report, which serves to determine the strategy they used as well as the number of landmarks they used (e.g., “I used the rock and the tree to find the objects”) and noticed (e.g., “I saw a mountain but I did not use it”). **b** There was a negative correlation between olfactory identification and the average number of navigational errors made in Part 2 of the learning trials (*r* = −0.37, Bootstrap BCa 95% CI [−0.52, −0.21]). **c** When we looked within navigation strategies, we found that this effect was driven by spatial learners (left) (*r* = −0.57, Bootstrap BCa 95% CI [−0.77, −0.33]), while response learners did not show such a relationship (right) (*r* = −0.22, Bootstrap BCa 95% CI [−0.49, 0.14]). Thus, it appears that the relationship between olfactory identification and navigation is specific to those who use a hippocampal-dependent navigation strategy. In other words, olfactory identification correlates with spatial learning, but not with stimulus-response learning

Next, we investigated whether olfactory identification was specifically related to spatial memory, or whether it was associated with other types of navigation, in this case stimulus-response learning. We looked at performance on the 4/8 VM (Fig. 2a) and found, at the group level, a significant negative correlation between olfactory identification and average navigational learning errors made on the 4/8 VM (*r* *=* −0.37, Bootstrap BCa 95% CI [−0.52, −0.21]) (Fig. 2b). This indicates that the greater the ability to identify odors, the fewer errors were made while learning the location of the objects, a finding consistent with the wayfinding results. When we looked at those who used a spatial memory strategy ('spatial learners') and those who used a stimulus-response strategy ('response learners') separately, we found that the correlation was driven by the spatial learners. Spatial learners (*n* = 24) showed a strong and significant negative association between olfactory identification and average navigational learning errors (*r* = −0.57, Bootstrap BCa 95% CI [−0.77, −0.33]) (Fig. 2c, left), while response learners (*n* = 33) did not show this association (*r* = −0.22, Bootstrap BCa 95% CI [−0.49, 0.14]) (Fig. 2c, right). To ensure that the lack of an association in response learners was not due to a ceiling effect, as their average navigational learning errors was lower than that of spatial learners (mean difference = –0.90, Bootstrap BCa 95% CI [−1.39, −0.39]), we conducted the same analysis based on a trial where spatial and response learners did not significantly differ in the number of errors they made. While spatial and response learners significantly differed in the first trial (mean difference = −2.52, Bootstrap BCa 95% CI [−3.49, −1.47]), they did not significantly differ on the second trial of the 4/8 VM (mean difference = 0.13, Bootstrap BCa 95% CI [−0.46, 0.75]). We proceeded to calculate the correlation between olfactory identification and the number of navigational learning errors made on the second trial. Within spatial learners, there was a significant correlation (*r* = −0.61, Bootstrap BCa 95% CI [−0.81, −0.24]), whereby better olfactory identification was associated with fewer navigational learning errors, while response learners showed no such correlation (*r* = −0.18, Bootstrap BCa 95% CI [–0.59, 0.33]. Thus, this analysis shows that the initial effect we found between olfactory identification and spatial memory is not due to a ceiling effect in response learners. Together, these results argue that the association between navigation and olfactory identification is only present in those who use hippocampus-dependent navigation strategies.

### mOFC thickness predicts olfaction and spatial memory

We then investigated the gray matter structure of two brain regions shown to be involved in olfactory identification and spatial memory in separate reports: the mOFC and hippocampus. We also included the caudate nucleus as a control region.

**Fig. 3:**
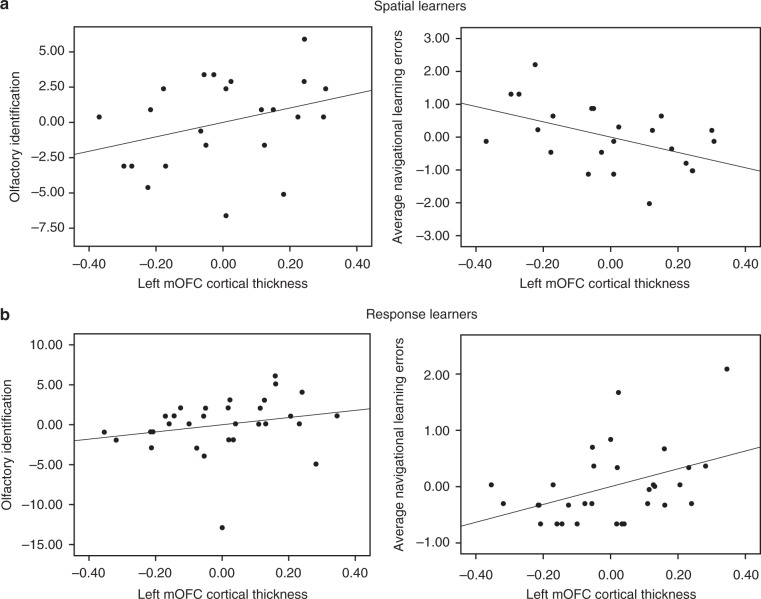
Left medial orbitofrontal cortex (mOFC) cortical thickness correlates with both olfactory identification and spatial memory. **a** We measured mOFC cortical thickness using CIVET. Within spatial learners, left mOFC cortical thickness correlated positively with olfactory identification (left) (*r* *=* 0.33, Bootstrap BCa 95% CI [0.02, 0.64]), and correlated negatively with average navigational learning errors (right) (*r* *=* −0.49, Bootstrap BCa 95% CI [−0.69, −0.24]). **b** Within response learners, left mOFC cortical thickness did not correlate with olfactory identification (left) (*r* = 0.23, Bootstrap BCa 95% CI [−0.07, 0.59]). Interestingly, it did correlate positively with average navigational learning errors (right) (*r* = 0.42, Bootstrap BCa 95% CI [0.13, 0.67]), which indicates that the mOFC may interfere with stimulus-response learning. These results indicate that the relationship between olfactory identification, navigation, and mOFC cortical thickness is specific to those who use a hippocampal-dependent navigation strategy (spatial memory)

In terms of the mOFC, spatial learners (*n* = 23) exhibited a significant positive correlation between left mOFC cortical thickness and olfactory identification (*r* *=* 0.33, Bootstrap BCa 95% CI [0.02, 0.64]) (Fig. 3a, left) and a significant negative correlation between left mOFC cortical thickness and average navigational learning errors (*r* *=* −0.49, Bootstrap BCa 95% CI [−0.69, −0.24]) (Fig. 3a, right). These results indicate that greater mOFC cortical thickness is associated with both fewer errors during spatial learning and better olfactory identification. In response learners (*n* = 31), there was no significant correlation between left mOFC cortical thickness and olfactory identification (*r* *=* 0.23, Bootstrap BCa 95% CI [−0.07, 0.59]) (Fig. 3b, left). Interestingly, left mOFC cortical thickness was positively associated with average navigational learning errors (*r* *=* 0.42, Bootstrap BCa 95% CI [0.13, 0.67]) (Fig. 3b, right). By way of its dense connections with the hippocampus, the mOFC may promote function of the hippocampus, which has been shown to interfere with stimulus-response learning^10,27,28^. In terms of right mOFC cortical thickness, there were no significant correlations with olfactory identification in either spatial learners (*r* *=* 0.24, Bootstrap BCa 95% CI [−0.15, 0.57) or response learners (*r* *=* 0.14, Bootstrap BCa 95% CI [−0.16, 0.53]. Average navigational learning errors, on the other hand, did significantly correlate with right mOFC cortical thickness in both strategy groups, albeit in different directions. In spatial learners, this correlation was negative, in that greater cortical thickness was associated with fewer errors (*r* *=* −0.49, Bootstrap BCa 95% CI [−0.70, −0.16], consistent with left mOFC results. In response learners, the correlation was positive (*r* *=* 0.34, Bootstrap BCa 95% CI [0.05, 0.60]), indicating an interference effect, as was seen with the left mOFC. Thus, as hypothesized, greater mOFC cortical thickness covaries positively with both navigational learning and olfactory identification within spatial learners. This was true for the left mOFC. While the right mOFC also showed a positive association with navigational learning within spatial learners, it did not show a relationship with olfactory identification.

### Hippocampal volume predicts olfaction and spatial memory

**Fig. 4:**
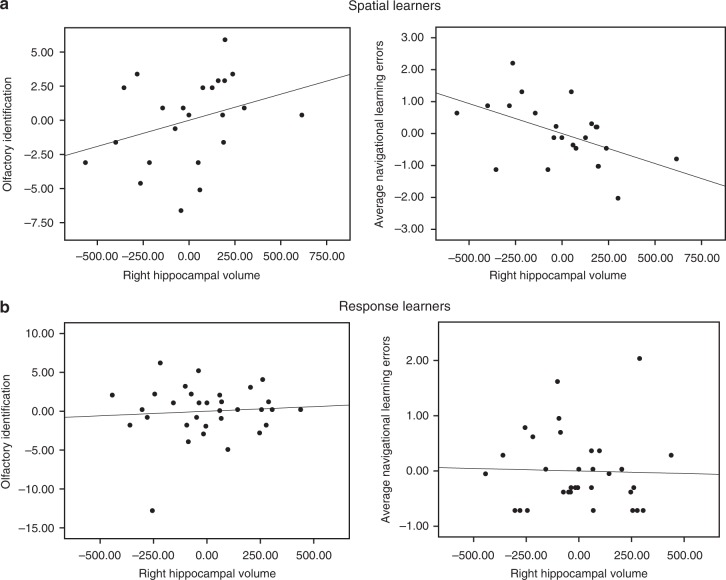
Right hippocampal volume correlates with both olfactory identification and spatial memory. **a** We measured hippocampal volumes using MAGeT-Brain. Within spatial learners, there was a positive correlation between right hippocampal volume and olfactory identification (left) (*r* = 0.32, Bootstrap BCa 95% CI [0.03, 0.60]), and a negative correlation between right hippocampal volume and average navigational learning errors (right) (*r* = −0.51, Bootstrap BCa 95% CI [−0.75, −0.20]). **b** Within response learners, right hippocampal volume did not significantly correlate with olfactory identification (left) (*r* = 0.08, Bootstrap BCa 95% CI [−0.32, 0.35]), nor with average navigational learning errors (right) (*r* = -0.03, Bootstrap BCa 95% CI [−0.42, 0.28]). These results indicate that the relationship between olfactory identification, navigation, and hippocampal volume is specific to those who use a hippocampal-dependent navigation strategy (spatial memory)

We hypothesized that, similar to the mOFC results, hippocampal volume would show an association with olfactory identification and navigational learning within spatial learners but not response learners. In spatial learners (*n* = 23), there was a significant positive correlation between right hippocampal volume and olfactory identification (*r* = 0.32, Bootstrap BCa 95% CI [0.03, 0.60]) (Fig. 4a, left) and a significant negative correlation between right hippocampal volume and average navigational learning errors (*r* = −0.51, Bootstrap BCa 95% CI [−0.75, −0.20]) (Fig. 4a, right). Response learners (*n* = 32) did not show these associations: there were no correlations between right hippocampal volume and olfactory identification (*r* = 0.08, Bootstrap BCa 95% CI [−0.32, 0.35]) (Fig. 4b, left), or average navigational learning errors (*r* = −0.03, Bootstrap BCa 95% CI [−0.42, 0.28]) (Fig. 4b, right). At the whole-group level, combining spatial and response learners, there were non-significant correlations between right hippocampal volume and olfactory identification (*r* *=* 0.17, Bootstrap BCa 95% CI [−0.09, 0.38] and between right hippocampal volume and average navigational learning errors (*r* *=* −0.30, Bootstrap BCa 95% CI [−0.57, 0.03]. In terms of the left hippocampus, there were no significant correlations with olfactory identification in either spatial learners (*r* = 0.11, Bootstrap BCa 95% CI [−0.22, 0.46]) or response learners (*r* = 0.09, Bootstrap BCa 95% CI [-0.29, 0.37]). Similarly, there were no significant correlations between left hippocampal volume and average navigational learning errors in either spatial learners (*r* = −0.38, Bootstrap BCa 95% CI [−0.70, 0.10]) or response learners (*r* = −0.11, Bootstrap BCa 95% CI [−0.40, 0.12]). These analyses indicate that greater volume of the right hippocampus is associated with better olfactory identification and fewer navigational learning errors within spatial learners. In accordance with our hypotheses, only those who relied on hippocampus-dependent navigation strategies (spatial memory) exhibited an association between navigational learning, olfactory identification, and hippocampal volume.

The control region, the caudate nucleus, showed no significant associations with olfactory identification nor with navigational learning within either strategy group. In spatial learners, there was no significant correlation between caudate nucleus volume and either olfactory identification (left caudate: *r* *=* 0.28, Bootstrap BCa 95% CI [−0.20, 0.63]; right caudate: *r* *=* 0.30, Bootstrap BCa 95% CI [−0.19, 0.64]), or average navigational learning errors (left caudate: *r* *=* −0.28, Bootstrap BCa 95% CI [−0.65, 0.13]; right caudate: *r* *=* −0.32, Bootstrap BCa 95% CI [−0.66, 0.07]). The results were the same in response learners: there was no significant association between caudate nucleus volume and either olfactory identification (left caudate: *r* *=* −0.21, Bootstrap BCa 95% CI [−0.12, 0.52]; right caudate: *r* *=* −0.21, Bootstrap BCa 95% CI [−0.17, 0.60]), or average navigational learning errors (left caudate: *r* *=* 0.00, Bootstrap BCa 95% CI [−0.36, 0.37]; right caudate: *r* *=* −0.003, Bootstrap BCa 95% CI [−0.38, 0.38]). These control analyses serve to support the specificity of the connection of the mOFC and hippocampus with olfactory identification and spatial memory.

### Lateralization of olfaction and spatial memory

We found effects in the right hippocampus and left mOFC. The right lateralization of the hippocampus for spatial memory and olfactory identification is consistent with the literature^12,14–16,24,29–34^. This right hippocampal lateralization is unlikely to be due to an unspecific effect of the right hemisphere as there were no effects found with the right caudate nucleus. In terms of the mOFC, an effect is also usually found in the right hemisphere with regards to olfaction. While we found an association between spatial learning and both the right and left mOFC, only the left mOFC showed an association with olfactory identification in spatial learners. There have been reports of left orbitofrontal cortex involvement in olfaction^12,32,35^ as well as reports showing no association between left or right orbitofrontal cortex and olfactory identification^14,35–37^. Therefore, the lateralization of the orbitofrontal cortex in olfactory function and more specifically in olfactory identification may not be as clear-cut as previously suggested in the literature. The discrepancies between studies are likely due to the methods used, including the olfactory identification tasks, brain imaging methods, and participant cohorts.

### Episodic and semantic memory do not mediate this association

Olfactory identification has been proposed to involve both episodic and semantic memory processes^12,38^. To verify whether an underlying episodic or, less likely, semantic memory mechanism is not responsible for the association between spatial memory and olfactory identification, we correlated olfactory identification with performance on the Rey Auditory Verbal Learning Task (RAVLT)^39^, an episodic memory task, and the Verbal Fluency test of the Delis–Kaplan Executive Function System^40^, a semantic memory task. There was no significant association between olfactory identification and performance on the RAVLT (total recall: *r* *=* 0.12, Bootstrap BCa 95% CI [−0.14, 0.37]; delayed recall: *r* *=* 0.003, Bootstrap BCa 95% CI [−0.29, 0.30]). There was also no significant association with the Verbal Fluency test (all Bootstrap BCa 95% CI crossed 0), consistent with a study that showed that older adults exhibited impairments in olfactory identification but not semantic memory^41^. Thus, these results suggest that olfactory identification and spatial memory are not spuriously correlated because of a shared underlying episodic or semantic memory component.

In order to assess whether other neuropsychological factors could explain our results, we compared spatial and response learners’ performance in various cognitive domains. Specifically, there was no between-group difference in performance on a battery of standard neuropsychological tests which included the RAVLT^39^, the Rey-Osterrieth Complex Figure^42^, and the Test of Non-verbal Intelligence-3^43^ (all Bootstrap BCa 95% CI crossed 0).

### Olfaction and spatial memory critically rely on the mOFC

**Fig. 5:**
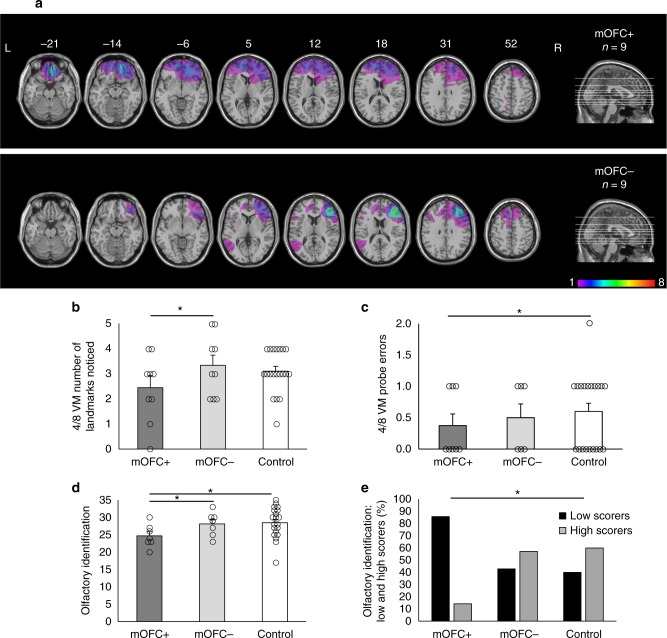
Patients with medial orbitofrontal cortex (mOFC) damage show reduced spatial memory. **a** Axial slices and midsagittal view of the ICBM52 MNI brain depicting the extent of the lesion overlap for patients whose frontal lobe lesions encompass the mOFC (mOFC + ; top) and patients whose frontal lobe lesions do not encompass the mOFC (mOFC-; bottom). The numbers above the axial slices indicate their z coordinates in MNI space. The colors indicate the degree to which lesions overlap within each group, as indicated by the color scale, which ranges from 1 to 8 patients. **b** In the 4/8 VM, the mOFC + group noticed fewer landmarks in the environment compared to the mOFC- group (mean difference = −1.17, Bootstrap BCa 95% CI [−2.20, −0.27]). **c** The mOFC + group also made fewer errors on the probe trial compared to the control group (mean difference = −0.24, Bootstrap BCa 95% CI [−0.55, −0.02]). **d** The mOFC + group exhibited worse olfactory identification than both the mOFC- group (mean difference = −4.99, Bootstrap BCa 95% CI [−8.54, −1.09]) and the control group (mean difference = −3.70, Bootstrap BCa 95% CI [−6.02, −1.12]). **e** We categorized participants as low (black bars) or high (gray bars) scorers on the olfactory indentification task according to the median across all groups (median = 27.5). The mOFC + group included more low scorers than the control group, *p* < 0.05 (Fisher’s exact test). These results indicate that mOFC damage affects both spatial memory and olfaction, as it is is associated with a lesser reliance on landmarks and worse olfactory identification. Error bars indicate the standard error of the mean. *indicates significance based on bootstrapped bias-corrected 95% confidence intervals. L Left, R Right

Overall, our neuroimaging results are consistent with work in patients with medial temporal lobe damage, who exhibit deficits in both olfaction^17,18,22,23^ and spatial memory^16,19–21^, demonstrating a critical role of the medial temporal lobe, and likely the hippocampus, in both processes. Our findings are also consistent with work in patients with mOFC damage, who present with olfactory deficits^18,24,25^. However, whether mOFC damage also yields spatial memory deficits has not yet been reported. In a second study, we directly tested whether mOFC is critical for both spatial memory and olfactory identification. In rodents, orbitofrontal cortex neurons appear to be tuned to certain navigational characteristics. For example, some neurons in this or surrounding regions are path-selective^44^, encode odor-place associations^45^, and fire when the rat is in a specific location and expects a reward^46^. In primates, there is little evidence that the orbitofrontal cortex processes spatial information^47–49^. However, the tasks used in primate studies do not involve cognitive mapping or navigation, which may explain the discrepancy with rodent and human studies, whose tasks are much more similar in that respect. In healthy young adult humans, we showed that mOFC fMRI BOLD activity and gray matter is associated with spatial learning in a dual-solution navigation task^7^. In a lesion study, therefore, we investigated whether patients with frontal lobe damage that affects the mOFC (mOFC + group) exhibit both olfactory and spatial memory deficits, compared to patients with frontal lobe damage sparing the mOFC (mOFC- group) and demographically-matched control participants with no brain damage (Fig. 5a).

We first investigated wayfinding performance across groups, and found no difference between the groups (all Bootstrap BCa 95% CI crossed 0). This suggested that, if spatial memory impairments are present in the mOFC + group, that they may be subtle in nature and may not be adequately captured using a wayfinding task. The 4/8 VM allows us to investigate various facets of navigation, including strategy use, navigational learning, and landmark use. In the 4/8 VM, the mOFC + group noticed significantly fewer landmarks in the environment compared to the mOFC- group (mean difference = −1.17, Bootstrap BCa 95% CI [−2.20, −0.27]) (Fig. 5b). However, there was no significant difference between any of the groups in the reported number of landmarks used to learn the object locations (all Bootstrap BCa 95% CI crossed 0). In the probe phase, the mOFC + group made significantly fewer probe errors compared to the control group (mean difference = −0.24, Bootstrap BCa 95% CI [−0.55, −0.02]) (Fig. 5c), indicating that they relied less on landmarks, even though subjectively they reported no difference in terms of the number of landmarks they used. The groups significantly differed in their ability to reach the learning criterion (*p* < 0.05, Fisher’s exact test). Interestingly, pairwise comparisons showed that the mOFC + group learned normally compared to the control group (Bootstrap BCa 95% CI crossed 0). Therefore, the fact that the mOFC + group noticed fewer landmarks, described above, is not related to a learning impairment. The mOFC- group had more difficulty completing the 4/8 VM task than the control group (*p* < 0.05, Fisher’s exact test), as three mOFC- patients could not reach the learning criterion, compared to one patient in the mOFC + group and zero control participants.

The mOFC + group exhibited impaired olfactory identification compared to both the control group (mean difference = −3.70, Bootstrap BCa 95% CI [−6.02, −1.12]) and the mOFC- group (mean difference = −4.99, Bootstrap BCa 95% CI [−8.54, −1.09]) (Fig. 5d). We categorized participants as low or high scorers on the olfactory identification task based on their scores according to the median across the three groups (median = 27.5): those who scored below the median were categorized as low scorers and those who scored above the median were categorized as high scorers. When comparing the percentage of low and high scorers, the mOFC + group included significantly more low scorers (86%) than the control group (40%) (*p* < 0.05, Fisher’s exact test) (Fig. 5e).

### Control analyses

The three groups did not differ in age, education, lesion volumes (all Bootstrap BCa 95% CI crossed 0), or sex (*p* > 0.05, Fisher’s exact test) (Table 2). We also compared the three groups on the same battery of standard neuropsychological tests as in the neuroimaging study, namely the Rey Auditory Verbal Learning Task^39^, the Rey-Osterrieth Complex Figure^42^, and the Test of Non-verbal Intelligence-3^43^. The mOFC- group had a significantly lower non-verbal intelligence quotient than the mOFC + group (mean difference = −9.74, Bootstrap BCa 95% CI [−19.75, −0.36]) and the control group (mean difference = −13.37, Bootstrap BCa 95% CI [−24.25, −3.25]) (Table 2). The mOFC- group also presented verbal memory deficits, as evidenced by significantly lower performance on the total recall of the Rey Auditory Verbal Learning Test compared to the control group (mean difference = −12.56, Bootstrap BCa 95% CI [−20.52, −4.71]) (Table 2). The navigational learning impairments exhibited by the mOFC- group may therefore be due to global cognitive deficits.

In summary, in this lesion study, we showed for the first time that mOFC damage affects performance on both olfactory identification and spatial memory tasks. These deficits were not explained by general cognitive impairment, as patients with mOFC damage performed similarly to control participants on standard neuropsychological tests. This argues for a specific contribution of this frontal region to both olfactory identification and spatial memory.

## Discussion

In the current set of studies, we report for the first time that olfactory identification and spatial memory are related in humans. There was a positive relationship between olfactory identification and spatial memory but not stimulus-response navigation, across both studies. The association between olfactory identification and spatial memory may be explained, at least in part, by common neural substrates for these two processes, i.e. the hippocampus and mOFC.

In the literature, approximately half of the studies report that the hippocampus is associated with olfactory identification performance, while the other half reports no such involvement of the hippocampus (e.g., refs. ^12,15,37,41,50^). The fact that the hippocampus is not consistently associated with olfactory identification may reflect inter-individual variability in hippocampal involvement. Our finding that the correlation between olfactory identification and hippocampal volume is present among spatial learners but not response learners suggests that identifying people’s spontaneous navigation strategies is useful in capturing this inter-individual variability.

Besides the hippocampus, we investigated the role of the mOFC. Similar to the hippocampal volume results, we found that mOFC cortical thickness is associated with both olfactory identification and navigational learning within spatial learners but not response learners. We also demonstrate a critical role of the mOFC in both processes, as patients with mOFC damage exhibit impaired olfactory identification as well as reduced spatial memory, as they encoded less spatial information and relied less on landmarks. Thus, our findings are consistent with previous lesion studies that have shown the orbitofrontal cortex to be critical for olfaction^24^, and additionally provide new evidence for the mOFC’s critical role in spatial memory. Interestingly, patients with mOFC damage made fewer errors on the probe trial than the control group, which indicates that stimulus-response learning may have been facilitated. Supporting this, patients from this group who used a spatial strategy reported having incidentally learned the sequence of target paths. In concordance with this, we found in the young adult cohort that greater mOFC cortical thickness is associated with worse stimulus-response learning. Thus, when intact, the mOFC appears to interfere with stimulus-response learning, while when it is damaged, stimulus-response learning seems to be facilitated. This is consistent with the rodent literature, whereby lesioning the hippocampus or the fimbria-fornix, which are part of the spatial memory neural circuit, facilitates stimulus-response learning^10,51^. Our results show that the mOFC has a critical role not only in olfactory identification, but in spatial memory as well.

It would be of interest for future lines of study to investigate other olfactory processes such as olfactory recognition, discrimination, and threshold, and their relationship with spatial memory. The olfactory hypothesis does not discriminate among these distinct functions, and so we may hypothesize that these too would be associated with spatial memory. Similarly, it would also be of interest to investigate identification performance in other modalities, and whether there would be a relationship with spatial memory and its neural correlates. However, based on the fact that H.M., who had bilateral medial temporal lobe damage, did not exhibit visual or tactile identification impairments^17^, we hypothesize that the relationship seen between spatial memory and olfactory identification is in fact specific to the olfactory domain.

Taken together, our findings point to a close relationship between spatial memory and olfactory identification. We showed that these two processes covary in healthy young adults, that they correlate with right hippocampal volume, and that they are impaired in patients with damage affecting the mOFC. Thus, the behavioral relationship between olfactory identification and spatial memory is reflected in their shared neural substrates, which may have come about due to the parallel evolution of the olfactory and hippocampal systems^3^. While further research remains to be done, these findings bring evidence that is consistent with the suggestion that the original function of the olfactory sense may have been to support cognitive mapping and spatial memory.

## Methods

### Participants

Sixty healthy young adults between the ages of 18 and 35 were recruited through university classified ads and participated in the neuroimaging study. Demographic information and performance on neuropsychological tests are shown in Table 1. Inclusion criteria included right-handedness. People with a history of neurological or psychiatric disorders, including alcohol or drug abuse, or history of head trauma with loss of consciousness, were excluded. In the behavioral analyses in which we assessed the relationship between olfactory identification and navigation, 57 participants were included as three participants did not complete the virtual navigation tasks due to lack of time. Two participants did not undergo MRI scanning, also due to lack of time. Therefore, 55 participants (23 spatial learners, 32 response learners) were included in the volumetry analyses. One participant’s cortical thickness processing failed, and thus 54 participants (23 spatial learners, 31 response learners) were included in the cortical thickness analyses. In previous studies, effects were found with as few as 15 participants per group, so we expected to find an effect with groups of at least 20 participants. Informed consent was obtained from the participants in conformity with the ethics committee of the Douglas Mental Health University Institute. Participants received monetary compensation for their participation.

**Table 1:** Participant demographics, navigation strategies, and performance on neuropsychological tests

	Women/men	Age (years)	Education (years)^a^	Spatial/response^a^	TONI-3 IQ^a^	RAVLT^a^	ROCF^a^
Healthy young adults (*N* = 60)	29/31	22.9 (3.5)	16.3 (2.2)	24/33	113 (13.9)	12.5 (2.1)	25.8 (5.9)

Standard deviations are shown in parentheses

*TONI-3* test of non-verbal intelligence-3, *RAVLT* delayed recall of the Rey Auditory Verbal Learning Test, *ROCF* delayed recall of the Rey-Osterrieth Complex Figure.

^**a**^Information is missing for some participants

We recruited 18 patients with focal lesions to the frontal lobes from the Cognitive Neuroscience Research Registry at McGill University. Patients’ demographic information and performance on neuropsychological tests are shown in Table 2. Patients were tested a minimum of six months after their lesions occurred (median = 6.6 years; range: 1–48 years). No other criteria were used for inclusion in the study. However, if patients suffered damage to primary olfactory regions, such as the olfactory nerve and olfactory bulb, they were excluded from the olfactory analyses, as this damage is a confounding factor when investigating the role of the mOFC in olfactory identification. They were categorized *a posteriori* into mOFC-involved (mOFC + ) or mOFC-spared (mOFC-) groups by L.D., who was blind to task performance at the moment of categorization, based on their most recent clinical CT or MRI imaging. The anatomical landmarks for the mOFC included the ventromedial margin of the cerebral hemisphere on the medial edge, the medial orbital sulcus on the lateral edge, and a line was drawn from the posterior branch of the medial orbital sulcus to the medial portion of the subcallosal gyrus to delineate the caudal edge of the mOFC^52^. The mOFC + group included six women and three men; mean age = 56.56 ± 16.18; mean education: 14.509 ± 3.72. The mOFC- group also included six women and three men; mean age = 60.00 ± 6.71; mean education: 14.89 ± 2.93). We previously found differences between lesion groups of as few as five participants, so we expected significant results with groups of at least five participants. Lesion etiology in the mOFC + group included six cases of tumor resection, two cases of aneurysm rupture, and one case of stroke. In the mOFC- group, there were six cases of tumor resection and three cases of stroke. Three mOFC + patients and one mOFC- patient had a history of psychiatric disorders (anxiety and depression). Among them, at the time of testing, one mOFC + patient was depressed and another mOFC + patient had an anxiety disorder. At the time of testing, one mOFC + patient and one mOFC- patient were taking psychoactive medication—anti-depressants in both cases.

**Table 2:** Participant demographics, navigation strategies, and performance on neuropsychological tests

Group	Women/men	Age (years)	Education (years)	Spatial/response	TONI-3 IQ	RAVLT^a^	ROCF^a^	Lesion volume (cc)
mOFC + (*n* = 9)	6/3	56.6 (16.2)	14.5 (3.7)	6/3	106.1 (6.1)	53.2 (13.1)	18.2 (8.4)	46.3 (6 - 173)
mOFC- (*n* = 9)	6/3	60.0 (6.7)	14.9 (2.9)	3/6	97.1 (11.8)	44.3 (10.0)	19.2 (5.1)	34.7 (9 - 79)
Control (*n* = 20)	15/5	57.3 (11.0)	16.5 (3.4)	15/5	109.9 (18.8)	58.2 (5.9)	18.3 (6.2)	—

Standard deviations are shown in parentheses

*TONI-3* test of non-verbal intelligence-3, *RAVLT* total recall of the Rey Auditory Verbal Learning Test, *ROCF* delayed recall of the Rey-Osterrieth Complex Figure.

^a^RAVLT and ROCF scores are missing for some participants

Twenty control participants matched to the patients on age and education were recruited using local advertisement (15 women, 5 men; mean age = 57.30 ± 11.02; mean education: 16.48 ± 3.39). Six participants had a history of psychiatric disorders (anxiety and depression). Among them, one participant was depressed and one participant had an anxiety disorder at the time of testing. In terms of psychoactive medication, one participant was taking anti-depressants, one participant was taking anxiolytics, and one participant was taking both anti-depressants and anxiolytics at the time of testing. The incidence of psychoactive drug use was similar across the three groups (*p* > 0.05, Fisher’s exact test).

Two patients were excluded from the olfactory identification analyses due to structural damage to sensory regions of the olfactory system. Medical information in the patient database indicated that one mOFC + patient had a severed olfactory nerve and another mOFC + patient had damage to the olfactory bulb. Four additional patients provided incomplete data: one mOFC + patient and three mOFC- patients could not reach the learning criterion in the 4/8 VM. They understood the task instructions but were unable to learn the location of the objects. For this reason, their learning stage data were included but probe trial data was not available for these patients. One mOFC + patient, one mOFC- patient, and one control participant did not perform the wayfinding task due to lack of time.

All participants in the lesion study gave written informed consent in accordance with the McGill University Research Ethics Board and received monetary compensation for their participation.

### Lesion analysis

The lesions identified from each patient’s most recent clinical CT or MRI scan were traced onto the ICBM52 MNI template by a neurologist (L.K.F.) experienced in imaging analysis and blind to patients’ task performance, using MRIcro (www.mccauslandcenter.sc.edu/mricro/). The same software was used to create lesion overlap images for each group (Fig. 5a).

### Olfactory identification

We used the Monell Extended Sniffin’ Sticks Identification Test (MONEX-40) to assess olfactory identification^53^. The MONEX-40 consists of 40 felt-tip pens, each infused with an odor. Experimenters washed their hands with unscented soap prior to testing. Each pen is placed for one to two seconds under the participant’ nose as the participant inhales the odor. For each pen, participants are shown four words on a screen and identify the one associated with the presented odor. The pens were originally developed to yield at least 35% accuracy across participants (Lundström, personal communication). In the neuroimaging study, two pens yielded an overall accuracy of less than 35% (warm milk and honey) and were therefore excluded from the analysis. Potential reasons for low accuracy include degradation of the odor over time or unfamiliarity due to cultural differences.

Most of the participants in the lesion study were francophone. For this reason, the MONEX-40 was translated into French. The English and French versions yielded similar scores (mean difference = 1.18, Bootstrap BCa 95% CI [−5.41, 6.57]). In the lesion study, three pens were discarded based on low accuracy (warm milk, apple, and peanut). Scores were therefore based on 37 items.

### Wayfinding in a virtual town

This task involves moving through a virtual town with a dimension of about 151 m × 153 m (23,10 m^2^) in an exploration phase and a probe phase. The town has many streets and buildings, and there are eight target landmarks that are identified with signs (e.g., movie theater, school, stores; Fig. 1a). This task was developed with the Unreal Tournament 2003 development kit (Epic Games, Raleigh, NC).

Exploration phase: Participants were given a minimum of 20 min to visit the town and to learn the location of the eight target landmarks. This exploration phase allowed participants to learn the relationships between the various landmarks, i.e. to construct a cognitive map^4^. Participants were required to reach two criteria; they had to pass by each of the eight landmarks at least twice and to walk every street at least once before moving on to the probe phase. If they did not do so spontaneously, an experimenter prompted them to move to unexplored areas by giving step-by-step directions when participants happened to be close to the area that was not visited. These directions were minimal and far apart to encourage learning from self-directed exploration. The exploration phase ended when participants reached the two criteria, which prevented participants from using a stimulus-response strategy, as they were not allowed to visit the town extensively and therefore could not learn habitual routes between each of the landmarks.

Probe phase: There were eight probe trials, in which participants started in front of a landmark and were asked to take the most direct route to another landmark. They were told that speed is not important. However, unbeknownst to them, there was a five-minute limit to each trial, which was only reached in 3.51% of the trials. Each landmark served as a start and/or target destination only once so that the direct routes between them were relatively unfamiliar.

The dependent variables were the average path tortuosity (indirectness of a path), the percentage of target landmarks found, and the average path error for successful trials. Average path tortuosity was calculated by dividing the total path length in a given trial by the shortest possible path length for that trial. We considered that targets may have been found by chance due to the generous time limit of five minutes per trial. Therefore, we also calculated two measures that minimized the impact of chance. We used a path cut-off that equaled three times the ideal distance for any given trial. This cut-off serves to minimize the impact of trials in which target locations were found by chance on the percentage of target landmarks found and average path error. For example, if a participant reached the target landmark but traveled more than three times the ideal distance, then the path error for this trial was not taken into account in the calculation of the average path error because it was assumed that the target was found by chance. It was however taken into account when measuring the percentage of target landmarks found.

### 4-on-8 Virtual Maze (4/8 VM)

The 4/8 VM^5,6^ was adapted from a maze task used in rodents^10^. It was comprised of a radial maze (a central platform with eight paths branching from it, each ending with a pit at the bottom of a staircase), surrounded by a rich landscape with proximal and distal landmarks (Fig. 2a). This task was developed with the Unreal Tournament 2003 development kit (Epic Games, Raleigh, NC).

The task involved a learning phase and a probe phase. Each of those phases was divided into two parts: in the first part, only 4 of the 8 paths were open. Participants were instructed to visit the accessible paths, to retrieve the objects at the bottom of the pits. Once they retrieved all four objects, participants were given time to look around and memorize the location of the paths that led to the objects. When they were ready, they moved to Part 2. Here, all the barriers were removed, making all the paths accessible. Participants were instructed to visit the remaining arms in order to retrieve the objects. In other words, they must avoid the paths that they visited in Part 1.

Before moving on to the probe phase, participants had to reach the learning criterion by finding the objects in Part 2 without making errors in at least one of the learning trials. We administered a minimum of three and a maximum of eight trials. In Part 1 of the probe phase, four of the eight paths were open, just as in the learning phase. However, in Part 2, a wall was erected around the radial maze, blocking the landmarks from participants’ view. The probe score measured participants’ reliance on landmarks when retrieving the objects.

At the end of the task, we conducted a verbal report, where we asked participants to describe how they solved the task from beginning to end. We used a specific structured questioning procedure and asked participants to describe their method of navigation for the whole task. Based on their response concerning the first trial, we categorized each participant as either a spatial or a response learner. Participants were categorized as spatial learners if they reported using two or more landmarks to remember the location of the pathways containing the objects, and did not report using a sequence (e.g., “There was a pathway on the left of the blossom tree, one next to the mountain peak, on the right […]”). They were categorized as response learners if they reported using a sequence or pattern from a single starting position to learn the location of the target pathways (e.g., “I started from the pathway directly in front of me, and remembered ‘open, open, closed, closed, open, closed, open, closed”). This verbal report procedure has previously been shown to be a robust measure of participants’ spontaneous navigation strategies and was shown to be sensitive to hippocampal function and structure^5–7^. We also used the verbal report to determine how many landmarks participants noticed and how many landmarks they used on average throughout the learning trials.

Based on our previous work^5,6^, the primary dependent measures were probe errors, number of trials to criterion, and average navigational learning errors, defined as the average number of errors on Part 2 of the learning trials. Other dependent variables included the number of landmarks noticed and average number of landmarks used during the learning phase of the task.

A few modifications were made to the 4/8 VM for patients with frontal lobe damage. The original task involves reversal learning, as participants have to memorize a different set of paths in the second trial and have to visit the opposite set of paths in Part 2 compared to Part 1 (participants have to locate the unvisited paths). Patients with mOFC damage are known to have reversal learning deficits^54,55^. To ensure that any deficit would be due to spatial memory impairments and not reversal learning deficits, we made the following changes: (1) all the trials have the same reward contingency, and (2) participants must visit the same arms in Part 2 as in Part 1. Because some patients with brain damage have working memory deficits, we administered a short verbal report after each trial to ensure that participants accurately reported the navigation strategies that they used. The dependent variables were the same as for the original 4/8 VM.

### Neuropsychological tests

To ensure that spatial and response learners did not differ in various cognitive domains which may confound the results, we administered the Rey Auditory Verbal Learning Task^39^ to assess verbal episodic memory, the Rey-Osterrieth Complex Figure^42^ to assess visuo-spatial episodic memory, the Test of Non-verbal Intelligence-3^43^ to evaluate non-verbal intelligence, and the Verbal Flency test^40^ to assess semantic memory. The same tests were administered in the lesion study to assess cognitive differences between the three groups, with the exception of the Verbal Fluency test.

### MRI data acquisition

Participants underwent brain MRI on a 3 Tesla Siemens Trio scanner at the Douglas Cerebral Imaging Centre. Their heads were immobilized with support cushions. The session started with a localizer scan, followed by a 9-min MPRAGE anatomical scan. We used a three-dimensional gradient echo acquisition to collect 192 contiguous 1 mm T1-weighted images in the sagittal plane (TR = 2300 ms; TE = 2.98 ms; flip angle = 9; field of view = 256 mm²).

### Behavioral analysis

In the neuroimaging study, we conducted partial correlations between navigation variables (wayfinding and 4/8 VM) and olfactory identification scores, with sex included as a covariate. Sex affects both wayfinding ability (men tend to outperform women^56^) and olfactory identification (women tend to outperform men^53,57^). In all analyses, we used bootstrapped bias-corrected and accelerated 95% confidence intervals (Bootstrap BCa 95% CI) to account for deviations from parametric assumptions and to determine significance. Bootstrapping uses a sample dataset and simulates 1000 datasets by resampling from the sample dataset with replacement. Resampling methods are advantageous in that they inherently correct for multiple comparisons^58,59^. Additionally, confidence intervals are more informative as they are an estimation of the population’s true value, rendering them more accurate and more robust than *p* values^60^, properties that are boosted when using bootstrapping methods^58^. Another advantage of resampling methods is that they estimate Type I and Type II error rates more precisely than classical *p* value adjustment methods^59^ as the experimental data is compared with a chance distribution. Multiple comparison corrections are therefore unnecessary. Lastly, bootstrapping is a non-parametric method and thus does not require transforming the data in cases where the data is not normally distributed^59^. One-tailed confidence intervals were calculated when analyses were hypothesis-driven. The analysis was implemented in SPSS Statistics 20 (IBM). Correlation graphs (Figs 1–4) were produced with SPSS.

In the lesion study, task performance was compared for the three groups (mOFC + , mOFC-, and control participants) with an analysis of covariance. Age was included as a covariate as the groups had large standard deviations, and because aging has an impact on both spatial memory and olfactory identification^61,62^. TONI-3 IQ varied across the three groups, and so was also included as a covariate. Navigation strategies were used as a covariate in the analyses pertaining to the number of landmarks noticed and used as the proportions of spatial to response learners were unequal between groups (see Table 2), and as spatial memory strategies are associated with a greater number of landmarks noticed and used. For the analyses pertaining to the neuropsychological tests, only age was used as a covariate. We used bootstrapped bias-corrected and accelerated 95% confidence intervals (Bootstrap BCa 95% CI) to account for deviations from parametric assumptions and to determine significance. One-tailed confidence intervals were calculated when analyses were hypothesis-driven. We also compared the proportion of participants who reached the learning criterion on the 4/8 VM and the proportion of low and high scorers on the olfactory identification task using Fisher’s exact test. For hypothesis-driven analyses, one-sided tests were conducted and were deemed significant if they returned a *p* value below 0.05. The lesion overlap figure (Fig. 5a) was produced with MRIcro and the bar graphs (Fig. 5b-e) were produced with Excel (Microsoft, Redmond, WA).

### Cortical thickness analysis

Structural images in the neuroimaging study were processed using CIVET (Version 1.12) (http://www.bic.mni.mcgill.ca/ServicesSoftware/CIVET)^63^. Images were linearly transformed and registered to the ICBM152 nonlinear template. Following this, image inhomogeneities were corrected using N3 non-uniformity artifact correction^64^, at which point the images were segmented into gray matter, white matter, and cerebrospinal fluid using INSECT^65^. Gray and white matter surfaces were subsequently produced for each hemisphere. The distances between the white and gray matter surfaces were measured in native space using the t-link method^66^. Each participant’s cortical thickness data was then smoothed using a 30-mm full-width at half-maximum (FWHM) surface-based diffusion blurring kernel. The resulting gray and white matter surfaces were quality controlled. CIVET was unsuccessfully run in one participant (a response learner). Following this, the cortical thickness maps were parcellated using the DKT atlas^67^. SurfStat (http://www.math.mcgill.ca/keith/surfstat/) was used to extract the mean cortical thickness values for the DKT regions in MATLAB (Mathworks, Inc.). Finally, in SPSS, we conducted correlations between mOFC cortical thickness and behavioral measures, with sex as a covariate as men generally have larger brains than women and as sex impacts both navigation and olfaction.

### Volumetric analysis

We used the Multiple Automatically Generated Templates (MAGeT) Brain tool to measure right and left hippocampal volumes^68^ as well as right and left caudate nucleus volumes^69^ in the neuroimaging study. This multi-atlas segmentation tool segments the hippocampal subfields and caudate nucleus in a fully automated fashion, and was designed to use as input a small set of high-quality manually-segmented atlases. We preprocessed the structural scans using N4 intensity correction^70^ and applied a head mask before running MAGeT-Brain to aid registration. Hippocampal subfield and subcortical atlases^68,69^ were used to manually segment the hippocampus and caudate nucleus in a set of atlas brains. In MAGeT-Brain, a library of 21 templates, an optimal number for segmentation accuracy^68^, is used to bootstrap each participant’s segmentation. We chose the 21 templates by first segmenting all the samples using the five manually segmented brains and selecting the ones with the best segmentations as a means of identifying participant scans which best register to the manually segmented atlases. Non-linear atlas-to-template registration is used to segment and label each template, resulting in a unique delineation of the subfields for each individual template. The bootstrapping technique yields 105 candidate labels for each participant (5 atlases × 21 templates), which are then fused through voxel-wise majority vote to produce one final segmentation. The Automatic Normalization Tools (ANTS) registration technique was used for the non-linear registration (https://github.com/vfonov/mincANTS). We determined the total hippocampal volume for each hemisphere by calculating the sum of the five subfield volumes: CA1, CA2/CA3, CA4/dentate gyrus, subiculum, and stratum radiatum, lacunosum, and moleculare. We visually inspected each output segmentation for quality control, which all segmentations passed. MAGeT- Brain volume correlations were covaried with sex, as men generally have larger brains than women and as sex impacts both navigation and olfaction, using SPSS. Intracranial volume was not used as a covariate as it did not correlate with any of our behavioral dependent variables (all Bootstrap BCa 95% CI crossed 0).

## Data Availability

The data that support the findings of this study are available from the corresponding author upon reasonable request.
